# Comparative Analysis between Individual, Centralized, and Federated Learning for Smartwatch Based Stress Detection

**DOI:** 10.3390/jpm12101584

**Published:** 2022-09-26

**Authors:** Muhammad Ali Fauzi, Bian Yang, Bernd Blobel

**Affiliations:** 1 Department of Information Security and Communication Technology, Norwegian University of Science and Technology (NTNU), 2815 Gjøvik, Norway; 2 Medical Faculty, University of Regensburg, 93053 Regensburg, Germany; 3 eHealth Competence Center Bavaria, Deggendorf Institute of Technology, 94469 Deggendorf, Germany; 4 First Medical Faculty, Charles University Prague, 12800 Prague, Czech Republic

**Keywords:** stress detection, privacy, individual learning, centralized learning, federated learning, smartwatch, machine learning

## Abstract

Machine learning has been proven to provide good performances on stress detection tasks using multi-modal sensor data from a smartwatch. Generally, machine learning techniques need a sufficient amount of data to train a robust model. Thus, we need to collect data from several users and send them to a central server to feed the algorithm. However, the uploaded data may contain sensitive information that can jeopardize the user’s privacy. Federated learning can tackle this challenge by enabling the model to be trained using data from all users without the user’s data leaving the user’s device. In this study, we implement federated learning-based stress detection and provide a comparative analysis between individual, centralized, and federated learning. The experiment was conducted on WESAD dataset by using Logistic Regression as the classifier. The experiment results show that in terms of accuracy, federated learning cannot reach the performance level of both individual and centralized learning. The individual learning strategy performs best with an average accuracy of 0.9998 and an average F_1_-measure of 0.9996.

## 1. Introduction

In today’s busy world, stress has become an interesting issue in recent years, gaining awareness in many countries. Stress can be defined as a unique affective state that occurs when an individual considers that their perceived resources or ability cannot cope with the perceived demand of a stimulus [1]. The latest survey by Acas in 2019 [2] about stress and anxiety at work reported that about 66% of working people have experienced work-related stress in the last 12 months. Hospital employees, who in fact are very familiar with this issue, are also exposed to high levels of work-related stress [3,4,5].

Stress at a low level is acceptable or maybe even positive, also called eustress. However, prolonged stress can have a negative impact on our physical, mental, and emotional health. Many studies reported that stress has a significant impact on the development of hypertension and coronary artery disease, diabetes, asthma, etc. [6]. Moreover, excessive stress also harms the employee’s productivity, increases absenteeism, and plays a crucial role in mental illness development, such as generalized anxiety disorder and depression [7]. According to studies, in the hospital setting for example, a higher stress level is significantly correlated with low patient safety [8,9]. Another study also suggested that a higher stress level of hospital staff results in riskier cybersecurity practices [10]. These studies are in line with a prior study [11], reporting that stressed people will be slow in learning something new and may choose less profitable decisions.

Monitoring an individual’s stress level has many advantages. Knowing their own stress level can help them in staying aware and feeling more in control of their response to situations and knowing when it is time to relax or take some actions to treat it properly [12]. Furthermore, this monitoring can help to early diagnose mental illness and disorders. The most common way to assess a stress level is the use of questionnaires (e.g., Perceived Stress Scale [13], Perceived Stress Questionnaire [14], etc.). However, this method takes time, so it is not convenient to use every day for continuous monitoring. Another approach for determining stress levels is to measure stress-related physiological reactions using sensors. The smartwatch is one of the most suitable devices to perform this stress monitoring task, especially in the working environment. A smartwatch offers a number of built-in sensors that can be used for multimodal-based stress detection including blood volume pulse, electrodermal activity, skin temperature, accelerometer, etc. Unlike many wearable devices that have very low usability and are not convenient to wear during work (e.g., chest-worn devices, finger-placed galvanic skin response (GSR) sensors, etc.), the smartwatch is well known and has a high degree of social acceptance due to their ubiquity in everyday life [15,16].

There has been a remarkable success of machine learning (ML) technologies in empowering practical artificial intelligence (AI) applications, including in medical fields. Many prior studies have used multi-modal sensor data and machine learning methods to develop stress detection systems such as Decision Tree, K-Nearest Neighbors (KNN), Random Forest, and Logistic Regression [17,18,19,20]. Machine learning techniques generally need a sufficient amount of data for training to perform well. Therefore, to create a robust method, we need to collect sensor data from several users and collect them at a central server for processing. However, the uploaded medical data may contain individual privacy-related and sensitive information. Privacy breaches can happen if the central server is compromised. Furthermore, the leakage can also happen even when well-intentioned individuals, who have access to the server, share the data for legitimate purposes. As a result, a growing number of studies place attention on safeguarding private data in analysis processes. Federated learning (FL) can be the solution to this privacy challenge. FL works by allowing each data register to train models on separate, isolated datasets while only sharing the trained models, which do not contain any personal information. The registers then send their models to a central server for aggregating them to a single, integrated model. This process is repeated for a number of iterations until a high-quality model is produced. In this work, we implement FL-based stress detection and provide a comparative analysis between individual, centralized, and federated learning.

The remainder of this paper is organized as follows. The introduction part is given in Section 1. Dataset, features, learning strategies, and evaluation methods for the stress detection task are explained in Section 2. The results and discussion of this paper are described in Section 3 and Section 4, while conclusions are provided in Section 5.

## 2. Materials and Methods

### 2.1. Dataset

A public dataset called WESAD (Wearable Stress and Affect Detection) [17] was used in this study. The dataset was created in the lab by the Ubiquitous Computing research group at the University of Siegen, Germany, and was made public in 2018. The data came from 15 participants consisting of 12 males and 3 females. The demographic information of the participants in this dataset is displayed in Table 1.

The data in the WESAD study were acquired using an Empatica E4 smartwatch and a RespiBAN chest band at the same time during specified tasks designed to capture three different affective states: neutral, stress, and amusement. Only Empatica E4 data are used in this study because the focus of this work is on smartwatch sensors. The built-in sensors on the smartwatch are skin temperature (ST), accelerometers (ACC), electrodermal activity (EDA), and blood volume pulse sensors (BVP). Each individual had a data collection session of at least 36.5 min, which included the neutral position for approximately 20 min, the stress situation for 10 min, and the amusement situation for around 6.5 minutes. During the neutral position, the participants were sitting/standing and neutrally reading provided magazines. During the stress situation, the participants faced the Trier Social Stress Test (TSST) [21] to induce their stress, whereas during the amusement situation, the participants watched a set of funny video clips. The neutral and relaxation sessions were combined into one non-stress class for the stress detection task in this study so that the classification problem was binary (stress and non-stress).

### 2.2. Features

In this study, we employed all the sensors’ data on the smartwatch including ST, ACC, EDA, and BVP. To extract the features, the signal data were segmented by using a 60-second sliding window with a sliding step of 0.25 s following the recommendation by Kreibig et al. [22]. Furthermore, we constructed 6 different signals for each sensor’s data: the original signal; its first and second derivatives; and the transformed signal data using a Discrete Wavelet Transform (DWT) with the Haar wavelet at 3 different frequencies (1 Hz, 2 Hz, and 4 Hz). Wavelet transforms can catch both frequency and time information, while immediate changes in signals can be captured by the Haar wavelet [23]. For the ACC data, in addition to the 3-dimensional signal data (*x*, *y*, and *z*-axis that are represented by $ACC_{x}$, $ACC_{y}$, and $ACC_{z}$, respectively), we also calculated their magnitude ($ACC_{norm}$) using Equation (1). In total, we have used signals consisting of 6 ST signals, 24 ACC signals, 6 EDA signals, and 6 BVP signals as displayed in Table 2. In the last step, we extracted 10 statistical features using BioSPPy and Numpy libraries [24] in Python as displayed in Table 3. In total, 420 features were analyzed for this study.

(1) $$ACC_{norm}=\sqrt{ACC_{x}^{2}+ACC_{y}^{2}+ACC_{z}^{2}}$$

### 2.3. Learning Strategies

In this study, three learning strategies are compared: individual learning; centralized learning; and federated learning. All those learning strategies used Logistic Regression (LR) as the machine learning model. LR is selected due to its good performance in stress detection tasks [25,26,27]. LR also provides relatively low computational complexity, compared to Deep Neural Networks (DNN), for example. Thus, it does not need a device with high computational power. LR in this study is implemented using the Scikit-learn library [28].

#### 2.3.1. Individual Learning

**Figure 1:**
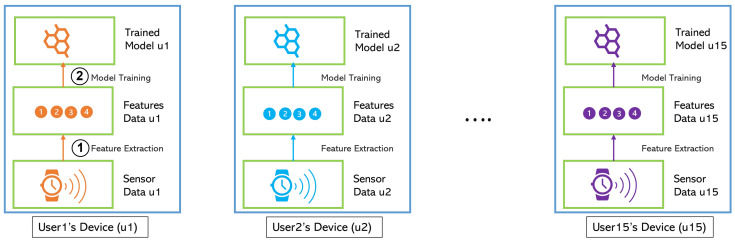
Individual Learning Scheme.

In this scheme, each user had their own model. As displayed in Figure 1, the user’s data never left their device. Using this scheme, the user’s device captured the sensor data, extracted the features, and then trained their individual machine learning model using their own data. In the end, each user attained a model personalized for them. Since there are 15 participants, there have been 15 separate models for each participant in this study. Like the raw sensor data, this model never left the user’s device and has never been shared with other users. The model will be used later on to detect the user’s stress. To be noted, this learning strategy needs a device that has enough computational power to perform the feature extraction and model training tasks.

This scheme offers a very high level of privacy because no data or model left the user’s device. Unlike the two other schemes, individual learning does not need a central server to combine the data or model, so it can minimize the cost. However, it prevents information sharing across users that generally can improve the performance of a machine learning model. In addition, if there is a new user, they cannot use the stress detection system right after the registration. The new user must collect their own stress data to train their individual model.

#### 2.3.2. Centralized Learning

**Figure 2:**
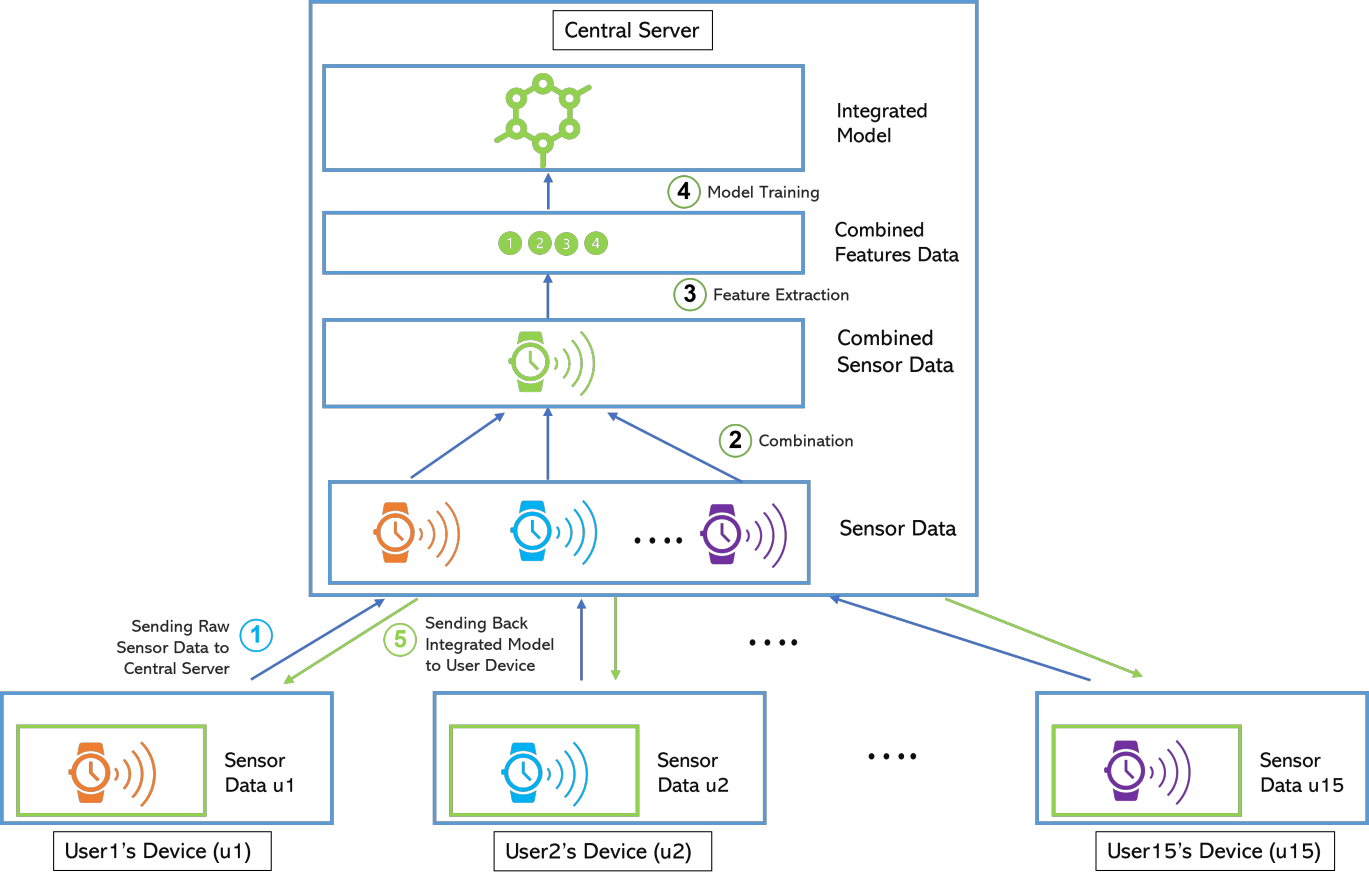
Centralized Learning Scheme.

In this scheme, we only have a single integrated model. Unlike individual learning, this learning strategy needs a central server to combine the data and train the integrated model. As shown in Figure 2, each user’s device captures the sensor data and then sends the raw data to the central server. Thereafter, the central server combines all the data from all users, extracts the features, and then trains a machine learning model using the combined data. As result, a single integrated model is created. This model is then sent to each user’s device and is used later to detect the user’s stress. Since the feature extraction and model training tasks are conducted on the central server side, this learning strategy does not need a device with high computational power. The user device only needs to do the stress detection/inference task using the model. Depending on the size of the dataset, training often takes several hours or more to complete. This stage of the process demands the greatest CPU or GPU power. The inference task on the other hand usually needs far less computing power than the training task. To minimize the computing power needed on the user’s device, the integrated model in this scheme can be stored on the server. When the user needs to perform the inference task on new data, the device can send the data to the server, and the server will detect the stress level of the data using the model and send the result back. However, this strategy requires the user’s device to be always online. If the integrated model is saved on each user’s device, the user’s device does not need to be online to predict the stress level.

This scheme offers a very low level of privacy because the user data leaves her/his device. This is sensitive data that can be used to disclose users’ personal information and their health status. However, it enables information sharing across users that generally can increase the robustness of a machine learning model. The other advantage of using this scheme is that a new user can use the stress detection system right after the registration by deploying the integrated model. The new user does not need to collect their own stress data and do the data labeling.

#### 2.3.3. Federated Learning (FL)

**Figure 3:**
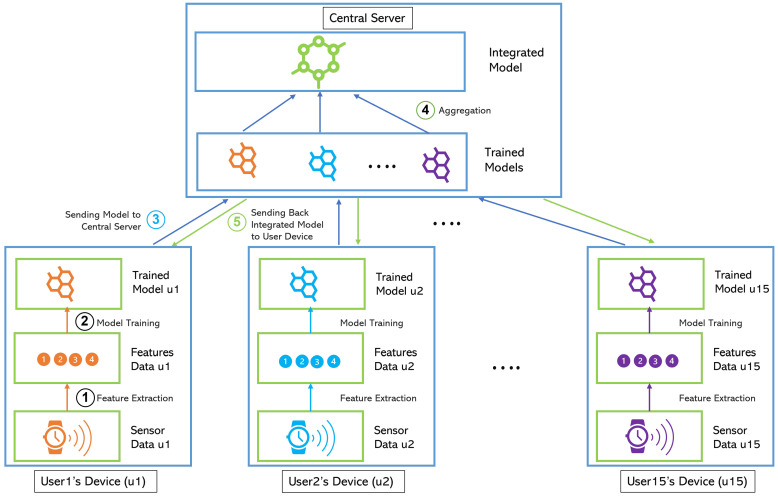
Federated Learning Scheme.

As displayed in Figure 3, the federated learning scheme is similar to centralized learning in terms of needing a central server and having just a single integrated model. The main difference between centralized and federated learning is that the user’s data will never leave the user’s device in federated learning, that way maintaining the user’s privacy. Federated learning in this study is implemented using Flower [29] with FederatedAveraging (FedAvg) aggregation strategy [30].

Stress data from sensors contain sensitive information that can be used to disclose users’ personal information and their health status. Therefore, the stress detection system needs to give more attention to privacy concerns. In Europe, the General Data Protection Regulation (GDPR) protects the users’ privacy by limiting the exchange of sensitive data [31]. On the other hand, the use of sensor data has many potential benefits. Therefore, a new family of privacy-preserving technologies is emerging to solve this problem. The goal of privacy-preserving technologies is to make the most of the data without jeopardizing users’ privacy. This technology employs strategies to reduce the amount of personal data held while maintaining the analysis operation. Several privacy-preserving methods have been proposed, and one of the techniques with high potential is Federated Learning.

Federated learning is a learning paradigm that aims to solve the problem of data privacy by collectively training algorithms without transferring data [30]. It has recently acquired popularity in healthcare applications [32,33]. FL allows for collaboratively using datasets without transferring the raw patient data outside of the institutions’ databases. As shown in Figure 3, each user’s device captures the sensor data and extracts the features. Furthermore, the machine learning model is trained locally on each user’s device. Next, the trained model is uploaded to the central server so that the central server can combine all the models and share the integrated model with each user’s device. This model will be used later to infer the user’s stress level. Some works show that models trained by FL can obtain performance levels comparable to those trained on centrally hosted data sets and exceeds models that only see isolated single-device data [34]. Successful implementation of FL could have a huge impact on enabling large-scale precision medicine, resulting in unbiased models while also respecting privacy issues [32]. To be noted, this learning strategy needs a device that has enough computational power to do the feature extraction and local model training tasks.

The federated learning scheme offers a very high level of privacy, because no data is leaving the user’s device. This scheme also enables information sharing across users that generally can improve the robustness of a machine learning model. In addition, if there is a new user, she/he can use the stress detection system right after the registration by using the integrated model without doing data collection first.

### 2.4. Evaluation

**Figure 4:**
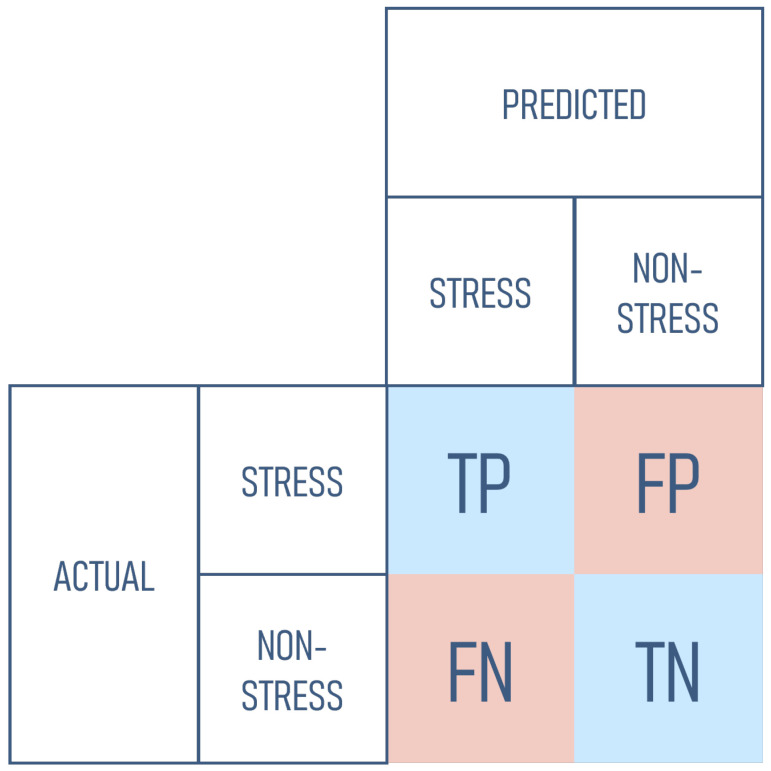
Confusion Matrix. Blue square means the data are correctly predicted while red square means the data are incorrectly predicted.

In this study, each data set is divided into two parts: training and testing data with a split ratio of 80:20. All the strategies use the training data for model training and testing data to evaluate the model performance. Several measurements including Accuracy (Acc), Precision (*P*), Recall (*R*), and F_1_-measure ($F_{1}$) were deployed for classifier performance evaluation. All measurements were calculated based on the confusion matrix displayed in Figure 4. True Positive (TP) and True Negative (TN) are the numbers of data that were correctly predicted. TP represents the number of stress data that were correctly predicted as stress, while TN represents the number of non-stress data that were correctly predicted as non-stress. Meanwhile, False Positive (FP), often called Type I Error, is the number of non-stress data that were incorrectly predicted as stress data, and False Negative (FN) or Type II Error represents the number of stress data that were incorrectly predicted as non-stress data.

The formulas for all measurements are displayed in Equation (2), Equation (3), Equation (4), and Equation (5) respectively.

(2) $$Acc=\frac{TP+TN}{TP+FP+FN+TN}$$

(3) $$P=\frac{TP}{TP+FP}$$

(4) $$R=\frac{TP}{TP+FN}$$

(5) $$F_{1}=2\frac{P\cdot R}{P+R}$$

## 3. Results

The results of stress detection using individual learning, centralized learning, and federated learning are presented in Table 4, Table 5 and Table 6. The experimental results show that individual learning is the most appropriate strategy for this task by obtaining an almost perfect performance with an average accuracy of 0.9998, an average precision of 0.9996, an average recall of 0.9996, and an average F_1_-measure of 0.9996. All individual models of the participants achieved 100% accuracy and F_1_-measure. Even the poorest individual model provided an accuracy of 0.9970 and F_1_-measure of 0.9951, which can still be considered almost perfect.

Meanwhile, centralized learning had also a good performance with an average accuracy of 0.9355, an average precision of 0.9125, an average recall of 0.8698, and an average F_1_-measure of 0.8783. The single integrated model from the centralized learning is excellent for inferring the stress level of most of the participants. The model achieved an accuracy below 0.9 just for three participants’ data (participant 5, 8, and 13). In terms of F_1_-measure, the model achieved a value below 0.9 for six participants’ data. The model best performed on the data of participant 10 with an accuracy of 0.9957, precision of 0.9880, recall of 0.9980, and F_1_-measure of 0.9930. In contrast, the worst result was gathered when detecting the stress level of participant 8 with an accuracy of 0.8545, precision of 0.9674, recall of 0.4771, and F_1_-measure of 0.6390.

Based on Table 6, federated learning had a relatively mediocre performance for the stress detection tasks in this study. It obtained an average accuracy of 0.8575, an average precision of 0.9892, an average recall of 0.5208, and an average of F_1_-measure of 0.6339. The integrated model from federated learning performed quite well on most of the participants’ data but performed very poorly on the data of some participants. This model achieved an F_1_-measure below 0.5 for 5 participants (participant 2, 4, 8, 9, and 13). The integrated model achieved the best result on the data of participant 3 with an accuracy of 0.9969, precision of 1.0000, recall of 0.9887, and F_1_-measure of 0.9943. On the contrary, the model performs the worst inferring the stress level of participant 4, with an accuracy of 0.7259, precision of 1.0000, recall of 0.0589, and F_1_-measure of 0.1113.

The study results suggest that the individual model achieved the best stress detection performance. This scheme outperformed both centralized learning and federated learning because it offers personalization by training the model separately for each user, using the user’s own data. The WESAD dataset labels the data based on the stimulus given to the participants. All the data recorded during the neutral and amusement condition, where the participants were reading magazines and watching funny videos, were labeled as non-stress, whereas all of the data recorded during the TSST session were labeled as stress. Different individuals will react to the stressors with varying intensity or duration [35]. Therefore, the personalized approach like the individual learning model surpasses the integrated model provided by centralized learning and federated learning. The integrated model aims at building a single model for all, so that it cannot adjust for each user.

These results also demonstrate that some models achieved quite good accuracy on some participants, but had a very poor F_1_-measure. To be noted, the stress dataset used in this study is imbalanced. It has more non-stress data than stress data. Therefore, accuracy is not good enough to be used as the evaluation measure. We need to perform the evaluation using precision, recall, and F_1_-measure. High accuracy means that the model can well predict the class. However, it is important to mention that accuracy is based on True Positive (TP) and True Negative (TN). In an imbalanced dataset where the number of non-stress data is higher than stress data, high accuracy may be achieved because the value of TN is very high even though the value of TP is very low. As an extreme example, if we have 100 testing data containing 90 non-stress data and 10 stress data and the model predicts all of the testing data as non-stress, the model will still get very good accuracy with 0.9. In this example, the model gets 90 TN and 0 TP. This model is actually not good because it cannot predict any stress data even though the accuracy is very high. In contrast with accuracy, the F_1_-measure of this model will be very low. Picking an example from the experimental result, the integrated model from federated learning applied to participant 4’s data achieved an accuracy of 0.7259, precision of 1.0000, recall of 0.0589, and F_1_-measure of 0.1113. The low recall with high precision means that the data predicted as stress by the model are very few, but most of the predicted labels are correct. In other words, this model mostly predicts the data as non-stress so that the TN value is very high, resulting in a high-value accuracy even though the TP value is very low because only a small amount of data were predicted as stress. In contrast with the accuracy, the F_1_-measure of this model is very low. Therefore, in an imbalanced dataset, F_1_-measure is a better measurement than accuracy.

## 4. Discussion

**Figure 5:**
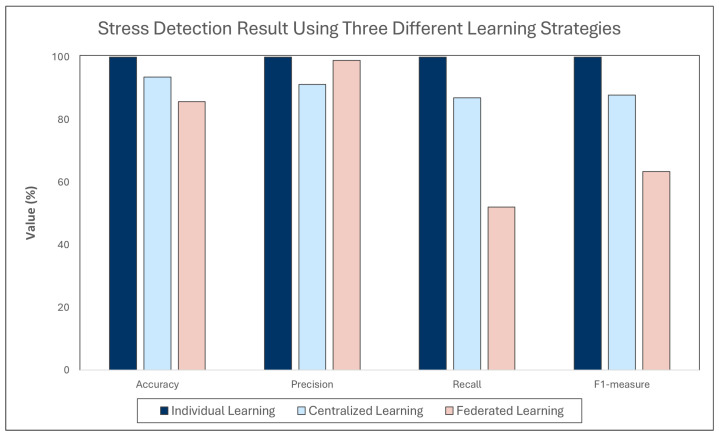
Stress Detection Results Using Three Different Learning Strategies.

This paper discusses the comparison of individual learning, centralized learning, and federated learning on the WESAD stress detection dataset. Generally, more data will make the machine learning model better and more accurate, because the more information we give to the model, the more it will learn and the more cases it will be able to correctly infer [36]. Therefore, integrated models such as centralized and federated learning are expected to be more accurate than individual learning. Surprisingly, the individual model surpasses in this study both the centralized and the federated learning as depicted in Figure 5. The WESAD dataset labels the data based on the stimulus given to the participants. Different participants may react differently to each stimulus. In this case, the personalized approach such as the individual learning model can adjust the model to the user’s behavior. The integrated model aims at building a single model for all so that it cannot adjust for each user. This study outcome is in line with another study about stress detection that also reported that a personalized model outperformed an integrated model [37].

Generally, federated learning is expected to perform worse than centralized learning. It is because centralized learning has direct access to all data while federated learning train the model locally and only communicates an updated model to a central server [38]. Surprisingly, the performance difference between the two strategies is very big. A more complex model such as Deep Neural Network (DNN) is needed to build a better federated learning model. Some previous work shows that federated learning with DNN can obtain performance levels comparable to those models trained using a centralized learning scheme [37,38]. Another study also suggested that less complex models perform worse than more complex models in federated learning [39]. However, a more complex model requires the user’s device to have a higher computational power to train the model. Additionally, a more complex model will also lead to higher communication costs between the user’s device and the central server. Thus, there will be a challenge to use a complex model for communication-sensitive applications [39].

Furthermore, since the WESAD dataset in this study is labeled based on the stimulus, there may be the possibility that the labels do not represent the participants’ actual stress levels. For example, during the TSST situation, there is the possibility that the participant was not feeling stressed (e.g., because they are good at public speaking) but all their gathered data during that session will be labeled as stress. Another issue could be that a participant was feeling stressed while watching the funny videos, because it reminded them of some traumatic events, for example, but all of their data during that session will be labeled as non-stress. Therefore, it will be of interest to see the comparison between the personalized and the integrated model on the stress dataset that is labeled based on the user’s subjective stress level measurement. In addition, the WESAD data collection was conducted in one session, which will make the data very similar. Thus, it is also of interest to see the comparison on the stress dataset, that is collected on multiple sessions to see how the model can perform across sessions.

Another factor that can also be considered is the usability of the three learning schemes for a new user. For centralized and federated learning, the new users can use the integrated model to predict their stress level right after the registration. For individual learning, however, the user must collect training data first. The users should record their data using the smartwatch during stress and non-stress condition. The users must also give the correct label to the data because the quality of the model heavily depends on the training data quality. This training data is used to train the personalized model for the users before they can infer their stress level automatically.

In addition, the computational cost is also different between these three schemes. Individual learning demands that a user’s device has enough computing power for feature extraction, model training, and stress detection tasks. Meanwhile, centralized learning requires less computing power for a user’s device, because all of the processes can be done on the central server. However, the device has to be always online since the device has to send the data to the central server. Federated learning needs a user’s device that has enough computing power to do the local training as well as a communication channel to exchange data between the device and the centralized server.

Finally, stress data are considered sensitive as they can be used to disclose the user’s health status. Based on a study on health data privacy, most of the interview subjects are worried about their data privacy on an individual level [40]. Therefore, the processing of this kind of data needs to pay more attention to privacy concerns. In centralized learning, all the data are collected on a centralized server. When these data are shared with the central server, privacy leaks can occur if the central server is compromised. Therefore, centralized learning can jeopardize users’ privacy. On the contrary, individual and federated learning strategies offer a high level of privacy. In federated learning, only the learning model, and no raw user data, is processed centrally. Meanwhile, individual learning provides a higher level of privacy as it does not require any user data or model to leave the user’s device.

## 5. Conclusions

In this study, the comparison between individual, centralized, and federated learning for smartwatch-based stress detection is discussed. In terms of accuracy, the individual learning strategy beats both centralized learning and federated learning. This is quite reasonable because different participants may react differently to stressors, so a personalized model is needed. The integrated model aims to build a single model for all so that it cannot adjust for each user. In terms of privacy, centralized learning requires all of the data to be shared with a centralized server. There is a risk of privacy breach, when the central server got compromised. In contrast, the individual learning strategy offers a very high level of privacy, since it does not require any user data or model to leave the user’s device. Federated learning also offers a high level of privacy, since only the learned model, and no raw user data, is processed in the central server. The only disadvantage of individual learning is the low usability for a new user. For centralized and federated learning, the new users can use the integrated model to infer their stress level right after the registration. In contrast, for individual learning, the users must collect training data first to build the personalized model.

In future work, a more complex model such as DNN can be used to improve the federated learning scheme performance. In addition, it will be interesting to see the comparison between individual learning, centralized, and federated learning on the stress dataset that is labeled based on the user’s subjective stress level measurement and collected on multi sessions, instead of only a single session.

## Figures and Tables

**Table 1:** Participants’ demographic characteristics in the WESAD dataset (N = 15).

Characteristic	Value, Mean (SD)
Age (years)	27.5 (2.4)
Height (cm)	177.6 (6.7)
Weight (kg)	73.1 (10.3)

**Table 2:** Signal data used in this study.

Sensor	Signal
Skin temperature (ST)	ST original signal
	ST first derivative signal
	ST second derivative signal
	ST signal with DWT with the Haar wavelet at 4 Hz
	ST signal with DWT with the Haar wavelet at 2 Hz
	ST signal with DWT with the Haar wavelet at 1 Hz
Accelerometers (ACC)	$ACC_{x}$ original signal
	$ACC_{x}$ first derivative signal
	$ACC_{x}$ second derivative signal
	$ACC_{x}$ signal with DWT with the Haar wavelet at 4 Hz
	$ACC_{x}$ signal with DWT with the Haar wavelet at 2 Hz
	$ACC_{x}$ signal with DWT with the Haar wavelet at 1 Hz
	$ACC_{y}$ original signal
	$ACC_{y}$ first derivative signal
	$ACC_{y}$ second derivative signal
	$ACC_{y}$ signal with DWT with the Haar wavelet at 4 Hz
	$ACC_{y}$ signal with DWT with the Haar wavelet at 2 Hz
	$ACC_{y}$ signal with DWT with the Haar wavelet at 1 Hz
	$ACC_{z}$ original signal
	$ACC_{z}$ first derivative signal
	$ACC_{z}$ second derivative signal
	$ACC_{z}$ signal with DWT with the Haar wavelet at 4 Hz
	$ACC_{z}$ signal with DWT with the Haar wavelet at 2 Hz
	$ACC_{z}$ signal with DWT with the Haar wavelet at 1 Hz
	$ACC_{norm}$ original signal
	$ACC_{norm}$ first derivative signal
	$ACC_{norm}$ second derivative signal
	$ACC_{norm}$ signal with DWT with the Haar wavelet at 4 Hz
	$ACC_{norm}$ signal with DWT with the Haar wavelet at 2 Hz
	$ACC_{norm}$ signal with DWT with the Haar wavelet at 1 Hz
Electrodermal activity (EDA)	EDA original signal
	EDA first derivative signal
	EDA second derivative signal
	EDA signal with DWT with the Haar wavelet at 4 Hz
	EDA signal with DWT with the Haar wavelet at 2 Hz
	EDA signal with DWT with the Haar wavelet at 1 Hz
Blood volume pulse sensors (BVP)	BVP original signal
	BVP first derivative signal
	BVP second derivative signal
	BVP signal with DWT and the Haar wavelet at 4 Hz
	BVP signal with DWT and the Haar wavelet at 2 Hz
	BVP signal with DWT and the Haar wavelet at 1 Hz

**Table 3:** Statistical Features.

No.	Features
1	Mean of the Signal
2	Minimum value of the signal
4	Maximum value of the signal
4	Median of the signal
5	Maximum signal amplitude
6	Signal variance
7	Standard signal deviation
8	Absolute signal deviation
9	Signal kurtosis
10	Signal skewness

**Table 4:** Individual Learning Result.

Participant	Acc	P	R	F${}_{1}$
1	1.0000	1.0000	1.0000	1.0000
2	1.0000	1.0000	1.0000	1.0000
3	1.0000	1.0000	1.0000	1.0000
4	1.0000	1.0000	1.0000	1.0000
5	1.0000	1.0000	1.0000	1.0000
6	1.0000	1.0000	1.0000	1.0000
7	1.0000	1.0000	1.0000	1.0000
8	1.0000	1.0000	1.0000	1.0000
9	1.0000	1.0000	1.0000	1.0000
10	1.0000	1.0000	1.0000	1.0000
11	1.0000	1.0000	1.0000	1.0000
12	0.9994	0.9980	1.0000	0.9990
13	0.9970	0.9960	0.9941	0.9951
14	1.0000	1.0000	1.0000	1.0000
15	1.0000	1.0000	1.0000	1.0000
**Average**	**0.9998**	**0.9996**	**0.9996**	**0.9996**

**Table 5:** Centralized Learning Result.

Participant	Acc	P	R	F${}_{1}$
1	0.9414	0.8250	1.0000	0.9041
2	0.9317	0.9809	0.7809	0.8696
3	0.9660	0.8916	1.0000	0.9427
4	0.9571	0.8716	1.0000	0.9314
5	0.8833	0.9658	0.5853	0.7288
6	0.9511	0.8726	0.9720	0.9196
7	0.9772	0.9827	0.9401	0.9609
8	0.8545	0.9674	0.4771	0.6390
9	0.9244	1.0000	0.7495	0.8568
10	0.9957	0.9880	0.9980	0.9930
11	0.9475	0.8540	0.9851	0.9149
12	0.9353	0.8812	0.9127	0.8967
13	0.8837	0.8575	0.7475	0.7987
14	0.9437	0.8400	1.0000	0.9130
15	0.9404	0.9098	0.8994	0.9046
**Average**	**0.9355**	**0.9125**	**0.8698**	**0.8783**

**Table 6:** Federated Learning Result.

Participant	Acc	P	R	F${}_{1}$
1	0.9131	0.8675	0.8089	0.8372
2	0.7565	0.9872	0.1670	0.2857
3	0.9969	1.0000	0.9887	0.9943
4	0.7259	1.0000	0.0589	0.1113
5	0.8511	1.0000	0.4447	0.6156
6	0.8700	1.0000	0.5484	0.7083
7	0.8578	1.0000	0.5227	0.6866
8	0.7796	1.0000	0.1835	0.3101
9	0.7820	1.0000	0.2781	0.4352
10	0.9390	0.9950	0.8016	0.8879
11	0.9524	1.0000	0.8337	0.9093
12	0.9097	0.9917	0.7123	0.8291
13	0.7620	1.0000	0.2288	0.3724
14	0.8880	0.9967	0.6232	0.7669
15	0.8778	1.0000	0.6110	0.7585
**Average**	**0.8575**	**0.9892**	**0.5208**	**0.6339**

## Data Availability

The source code for individual, centralized, and federated learning in this paper can be found at https://github.com/cahkanor/WESAD-Stress-Detection-Logistic-Regression, (accessed on 17 August 2022).

## Abbreviations

The following abbreviations are used in this manuscript:

ML	Machine Learning
AI	Artificial Intelligence
KNN	K-Nearest Neighbors
FL	Federated learning
LR	Logistic Regression
DNN	Deep Neural Networks
FedAvg	Federated Averaging
DWT	Discrete Wavelet Transform
WESAD	Wearable Stress and Affect Detection
ST	Skin temperature
ACC	Accelerometers
BVP	Blood Volume Pulse
EDA	Electrodermal Activity
GSR	Galvanic Skin Response
TSST	Trier Social Stress Test
CPU	Central Processing Unit
GPU	Graphics Processing Unit
GDPR	General Data Protection Regulation
Acc	Accuracy
*P*	Precision
*R*	Recall
TP	True Positive
TN	True Negative
FP	False Positive
FN	False Negative
